# Supplementary material for: Multiplexed 129Xe HyperCEST MRI Detection of Genetically Reconstituted Bacterial Protein Nanoparticles in Human Cancer Cells
Source: Contrast Media Mol Imaging. 2020 Mar 12;2020:5425934. doi: 10.1155/2020/5425934 (PMC7091528; doi:10.1155/2020/5425934)
Supplement: Supplementary Materials — Supplementary Text: we described how to choose gas vesicle genes for our study in the selection of GV genes section. Note on TEM observation of GV structures is the short technical comments to observe GVLP structures by TEM. The purified GVLP structure and size distribution section is the description on the variability of the size and shape of GVLP measured and observed by TEM and DLS in the study. Supplementary Figure 1: FACS data on the fluorescence level of GVLP-expressing stable cells. Each fluorescence indicated the expression level of gas vesicle genes. Supplementary Figure 2: DLS data on the size distribution of the purified GVLPs from the GVLP-expressing stable cells. Supplementary Figure 3: a Z-spectrum of GV_AC20 cells shown as a negative data on the HyperCEST contrast of GVLP expressed in the cells. Supplementary Figure 4: TEM data of the embedded ultrathin section of the GV_AC20 cells and putative GVLPs are indicated by a white arrow. Supplementary Figure 5: the figure points out the importance of glutaraldehyde treatment for TEM observation of purified GVLPs from the GV_AC28 cells. Supplementary Reference: the publication is cited in the selection of GV genes section of the Supplementary Text. [file 5425934.f1.docx]

Multiplexed ^129^Xe HyperCEST MRI detection of genetically-reconstituted bacterial protein nanoparticles in human cancer cells.

**Authors:** Ryota Mizushima, Kanako Inoue, Atsuko H. Iwane, Tomonobu M. Watanabe, Atsuomi Kimura,

**Supplementary Information**

1. Supplementary Text

2. Supplementary Figures 1-5

3. Supplementary Reference

**Supplementary Text**

**Selection of GV genes**

We chose the *gvpC28* gene from *planktothrix agardhii* instead of *planktothrix rubescens* in this study because the full sequence of the *gvpC28* gene was not available on the Genbank database. In addition, because previous studies indicated that a heterologous combination of GV genes could be utilized to induce GVs with altered size and shape into bacterial species ^1^, we assumed that the heterologous combination of GV genes derived from *planktothrix rubescens* and *planktothrix agardhii* could be used to produce GVLPs for these closely related strains.

**Notes on TEM observation of GV structures**

In an initial effort to confirm GVLP expression in the cells, we observed ultra-thin slices of embedded GV_AC20 cell samples by TEM and found the putative GVLPs (indicated white arrow in Supplementary Figure 4), exhibiting the characteristic bicone structure, although the complexity of other intracellular components made it difficult to undeniably confirm GV expression.

It is noteworthy that fixation of the GVLP purified from GV_AC28 cells with 2.5 % glutaralaldehyde (GA) prior to drying and staining was found to be essential for observation of distinct GV structures; without GA fixation, we could not observe distinct GV structures in our experiments (Supplementary Figure 5).

**Purified GVLP structure and size distribution**

The TEM image of GVLPs purified from GV_AC20 cells in Figure 2b shows that the width and length of GVLPs was heterogeneous and ranged from ~ 200 nm to 300 nm. GVLP shape appeared to be biconical or cylindrical without conical ends, comprising a linear outline. The observed cylinders without conical ends may indicate deficient structure formation (e.g. lack of closed protein shell structure); this would reasonably explain the discrepancy between observed TEM sizes (Figure 2b) and lower size distribution peak of about 90 nm in DLS data (Supplementary Figure 2). In contrast, TEM images of GVLPs purified from GV_AC28 cells indicated a more rounded outline compared to other GV cells. The GV size ranged from 100 nm to 700 nm, and included the largest GVs among all that were generated. The two peaks of GVLPs purified from GV_AC28 cells in Dynamic Light Scattering (DLS) data (Supplementary Figure 2) may corresponded to the relatively broad particle size distribution of GVLP in GV_AC28 cells and/or aggregation of the particles. The TEM data showed that the length of GVLPs purified from GV_AC16C20 cells was around 100 nm or less, with a width of 50 - 60 nm. DLS data of GVLPs purified from GV_AC16C20 cells indicated a relatively　homogeneous size distribution with a peak at ~ 80 nm, which is in accordance with the TEM data.

**Supplementary Figures**

**Supplementary Figure 1 - Cell population distribution against fluorescence level, analyzed using FACS and shown as histograms for each GV cell type.** The first, second and third column of histograms shows the fluorescence levels of GFP, mKate2 and mKO2, respectively.


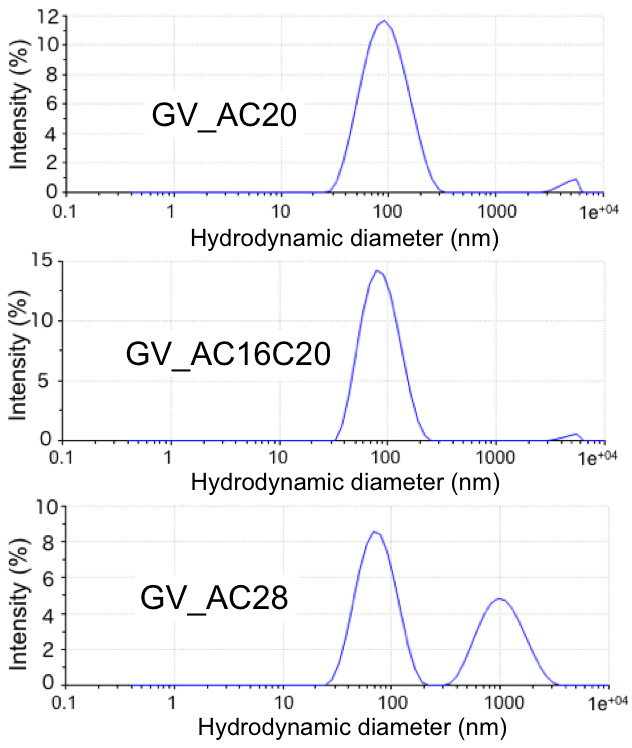


**Supplementary Figure 2 - Dynamic Light Scattering (DLS) data on the size distribution of each purified GVLP type.** Note that indicated sizes should be interpreted with caution, because GVLPs are non-spherical.


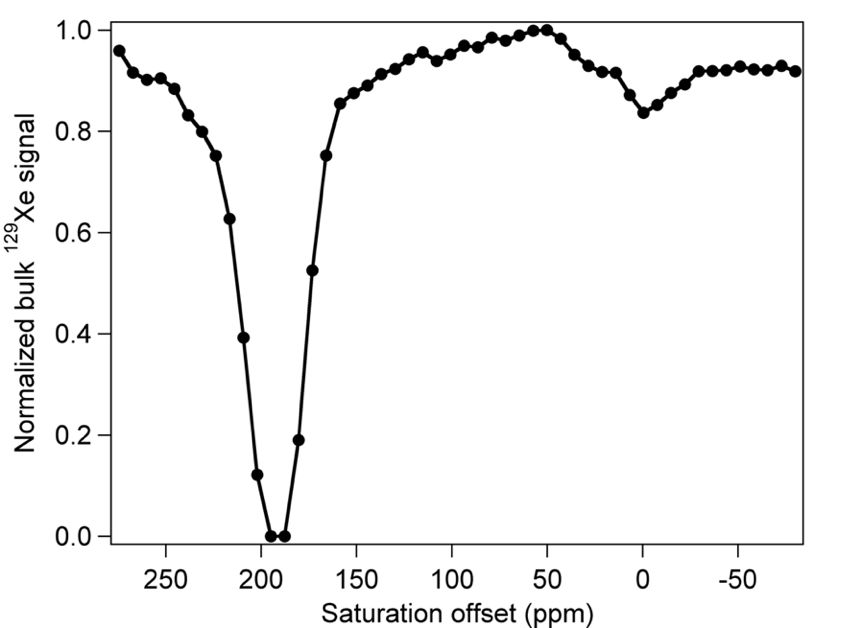


**Supplementary Figure 3** - **Z-spectrum of a GV_AC20 cell sample at 2 s saturation time with 8.0 x 10^7^ cells / ml.** (c.f. Figure 3 of main manuscript)


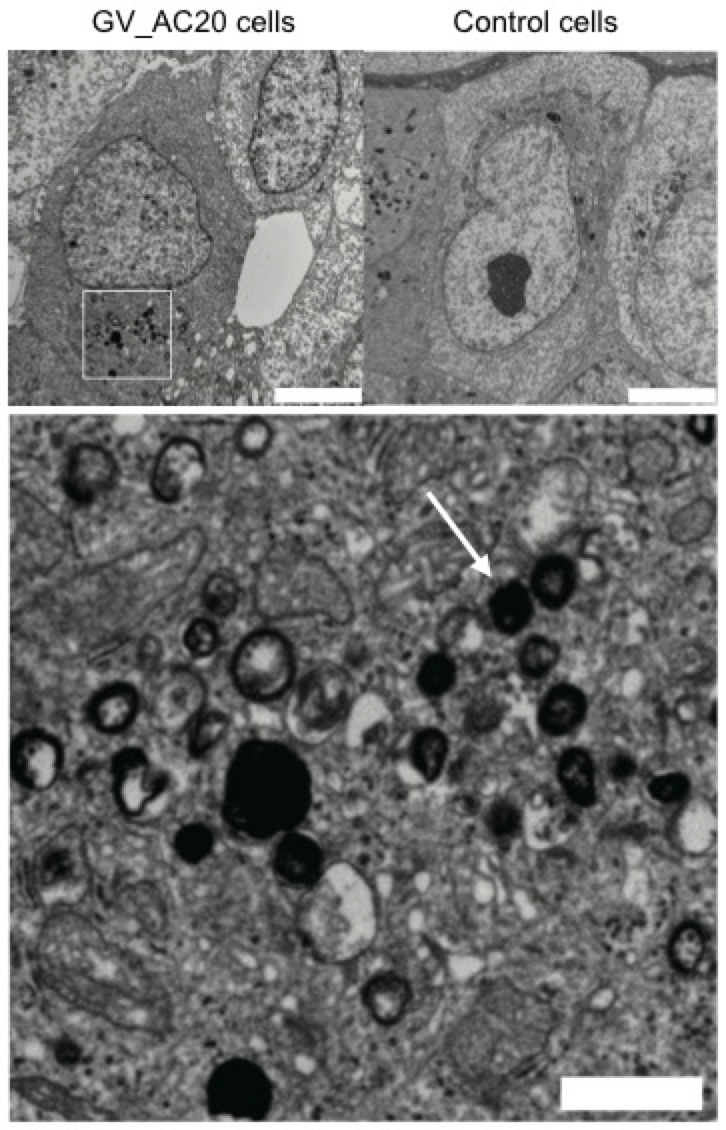


**Supplementary Figure 4 – TEM observation of ultra-thin sections of GV_AC20 cells.** Top: Sectional TEM images of embedded GV_AC20 (upper left) and control KPL-4 (upper right) cells are shown. Scale bars indicate 5 μm in both images. Bottom: Magnified region of interest (depicted by white square in upper left image) of the GV_AC20 cell image. The scale bar indicates 1 μm. The white arrow indicates a biconical gas vesicle.


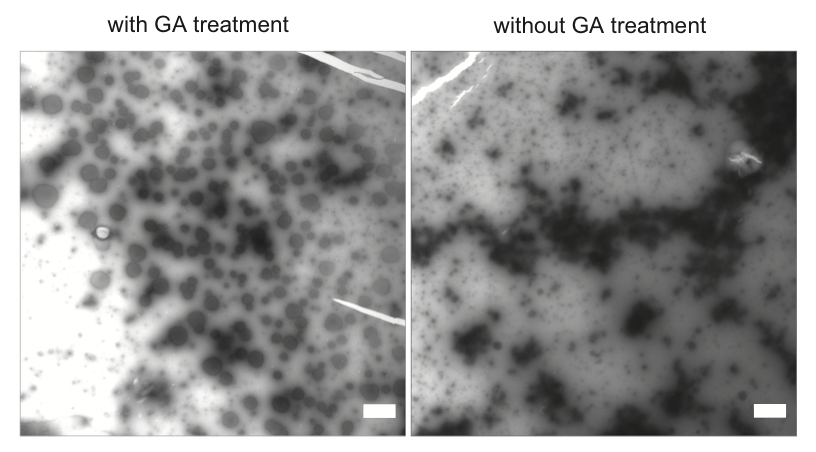


**Supplementary Figure 5 - TEM images of GVLPs purified from GV_AC28 cells with and without glutaraldehyde (GA) treatment prior to negative staining.** Scale bars indicate 1 μm in both images. These images illustrate the importance of the GA treatment for visualization of GV structures.

**Supplementary Reference**

1. Offner, S., Ziese, U., Wanner, G., Typke, D. & Pfeifer, F. Structural characteristics of halobacterial gas vesicles. *Microbiology* **144,** 1331–1342 (1998).
